# Probabilistic Clustering for Data Aggregation in Air Pollution Monitoring System

**DOI:** 10.3390/s25237285

**Published:** 2025-11-29

**Authors:** Vladimir Shakhov, Olga Sokolova

**Affiliations:** The Artificial Intelligence Research Center, Novosibirsk State University, 630090 Novosibirsk, Russia

**Keywords:** air quality monitoring, mobile sensor networks, artificial intelligence, unsupervised learning, smart clustering, expectation–maximization algorithm

## Abstract

Air pollution monitoring systems use distributed sensors that record dynamic environmental conditions, often producing large volumes of heterogeneous and stochastic data. Efficient aggregation of this data is essential for reducing communication overhead while maintaining the quality of information for decision making. In this paper, we propose an unsupervised learning approach for soft clustering of sensors in air pollution monitoring systems. Our method utilizes the Expectation–Maximization algorithm, which is an unsupervised machine learning method and probabilistic technique, to cluster sensors into distinct sets corresponding to normal and polluted zones. This clustering is driven by the need for a dynamic data transmission policy: sensors in polluted zones must intensify their operation for detailed monitoring, while sensors in clean zones can reduce reporting rates and transmit condensed data summaries to alleviate network load and conserve energy. The cluster membership probability enables a tunable trade-off between data redundancy and monitoring accuracy. The high efficiency of the proposed AI-based clustering is validated by the simulation results. Under common pollution scenarios and with adequate sample sizes, the EM algorithm exhibits a relative error below 5%. The presented approach provides a foundation for a wide range of intelligent and adaptive data aggregation protocols.

## 1. Introduction

Environmental degradation, particularly air pollution, ranks among the foremost threats to global health [1]. This crisis is primarily driven by the rapid expansion of urban industrial activity, a growing global transportation network, and large-scale burning of biomass, which together emit a complex mixture of hazardous particulate matter and gaseous pollutants. These insidious emissions significantly degrade air quality, leading to a marked increase in a wide range of diseases, from acute respiratory infections to chronic cardiovascular conditions and cancer. The most devastating impacts are concentrated in densely populated megacities, where intense emission sources and widespread human exposure converge with dangerous consequences. Consequently, the development and implementation of comprehensive, intelligent air quality monitoring (AQM) systems have become essential for public health protection. The key challenge lies not only in obtaining reliable data but also in ensuring its timeliness, broad geographic coverage, and continuous operation [2,3]. The data from these systems provide the essential foundation for effective environmental policies, proactive public health advisories, and rigorous regulatory measures. Ultimately, this systematic and technologically advanced approach is vital for safeguarding populations, guiding sustainable urban development, and securing a healthier quality of life for the millions of people in the world’s ever-expanding urban centers.

Traditional air pollution monitoring, which relies on fixed stations and temporary laboratories, is often hampered by high costs and an inability to provide dense spatial coverage. Modern systems overcome these limitations by deploying extensive networks of low-cost wireless sensors. A notable advancement is the integration of mobile sensors mounted on vehicles or drones, enabling dynamic, real-time data collection along transport routes and identifying pollution hotspots that fixed stations inevitably miss [4]. These pervasive sensor networks are a significant application of the Internet of Things (IoT), forming a core component of the smart city infrastructure [5]. Mobile air quality sensors transmit a continuous data stream to decision making centers, enabling the creation of highly detailed, real-time pollution maps. This capability facilitates intelligent urban management, including adaptive traffic control to reduce congestion-related emissions and the delivery of personalized air quality alerts to citizens. However, due to constraints from limited resources, these monitoring networks require careful optimization. A systematic review of the methods employed for this purpose is provided in [6]. An attractive optimization strategy is to cluster sensors into normal and polluted zones. This enables a dynamic transmission policy as follows. Sensors in polluted zones intensify operation for high-resolution data, while those in clean zones reduce reporting rates and send summaries to conserve energy and alleviate network load. A significant challenge, however, arises from the fuzzy and unstable boundaries of these clusters.

The distribution and concentration of air pollutants, including particles, volatile organic compounds, and microorganisms, are largely dependent on stochastic and highly dynamic meteorological factors [7,8,9]. Urban structure, specifically building density, green spaces, and the shape and size of buildings, contributes to air pollution patterns by either facilitating or inhibiting the dispersion of pollutants [10,11]. This is exemplified by the urban canyon effect, which is where tall buildings along narrow streets trap emissions, creating localized pockets of dangerously high pollution concentrations [12,13]. The concentration of pollutants like particulate matter (PM_2.5_), nitrogen dioxide (NO_2_), and ozone (O_3_) is characterized by pronounced spatiotemporal heterogeneity. Moreover, this variability is further compounded by vertical dynamics [14]. Therefore, from a geometric perspective, the spatiotemporal distribution of pollutant concentrations forms a heterogeneous and fluid patchwork. Transitions between “polluted” and “clean” zones can be undefined. Sensors located just a few meters apart can detect significantly different pollutant levels. This dynamic and stochastic nature of pollution makes it unrealistic to divide mobile sensors into strictly polluted and unpolluted clusters, rendering the use of hard clustering algorithms impractical.

This paper presents a probabilistic clustering framework for air quality sensor networks based on the Expectation–Maximization (EM) algorithm. The core practical contribution is a dynamic data transmission policy that utilizes soft cluster membership probabilities to intelligently allocate network resources. This approach achieves significant energy savings in clean zones by reducing transmission frequency and volume, while simultaneously enhancing monitoring resolution in polluted areas through intensified data collection. The intensification of operations in polluted zones enables high-frequency tracking of pollution dynamics and provides detailed data for source identification and analysis. Furthermore, an increased volume of data transmission ensures reliable delivery of critical environmental data [15]. Consequently, powering down sensors in clean zones becomes a strategically justified mechanism for energy conservation. Informed by the results from our prior work [16], this paper introduces an efficient and problem-specific realization of the EM clustering algorithm. To the best of our knowledge, this constitutes the first implementation of EM clustering in this context.

The rest of this paper is organized as follows. The paper proceeds with a review of related work in Section 2. Section 3 details the proposed methodology, providing a comprehensive description of the Expectation–Maximization algorithm, the specification of the probabilistic air pollution model, and the specific implementation of EM clustering. Section 4 presents a performance analysis, and Section 5 provides concluding remarks.

## 2. Related Work

The escalating challenge of urban air quality has intensified the focus on advanced pollution monitoring systems. Current research utilizes a heterogeneous mix of stationary reference stations, mobile laboratories, and pervasive networks of wireless sensors [16,17,18]. A foundational review in this domain [17] systematically evaluates the deployment of Wireless Sensor Networks (WSNs) for urban atmospheric sensing. It underscores the critical integration of geospatial data, IoT architectures, and solid-state sensor technologies. The analysis prioritizes key network performance metrics, including energy efficiency, nodal lifetime, packet delivery latency, and network throughput, which are paramount for sustainable large-scale deployment. Building on these principles, a practical implementation is demonstrated in [19], which details an industrial air quality monitoring system embedded within smart city infrastructure. This architecture co-opts the city’s street lighting grid to serve as a backbone for sensor placement and data backhaul. By utilizing streetlights as powered, elevated nodes, the system facilitates real-time data acquisition from distributed sensors, forwarding telemetry to aggregation points for processing and enabling dynamic public health alerts. The paper [20] presents a technical analysis of the latest advances in air pollution detection with a focus on air pollutants, sensor technologies, and IoT frameworks.

The review by [21] analyzes the application of advanced artificial intelligence (AI) in environmental research, covering both machine learning (ML) and deep learning (DL). It notes the current dominance of ML, highlighting the Random Forest method for achieving accuracies up to 98.2%. The study by [22] asserts that low-cost air monitoring sensors can achieve high effectiveness when paired with modern ML methods. The authors emphasize that while sensor accuracy depends on factors like gas sensitivity and environmental conditions, ML models can capture complex interdependencies in sensor responses to correct readings. Their research demonstrates that even simple models like multiple linear regression, when implemented on a microcontroller, significantly enhance the performance of low-cost CO, O_3_, and CO_2_ sensors. To enhance calibration model performance, a low-cost, multi-parameter air quality monitoring system utilizing various machine learning algorithms is presented in [23]. The work in [24] describes a methodology that employs machine learning to predict air quality. The approach first applies a decision tree algorithm to extract direct rules for real-time detection, followed by a process-mining algorithm to model changes in air conditions.

Optimizing data transmission in air pollution monitoring is essential for balancing system reliability with operational costs, primarily by reducing power consumption and network congestion [25,26]. This can be achieved through strategies such as optimized transmission scheduling, controlling the volume of transmitted data, reducing packet sizes, rational choice of the number of sensors, and adopting energy-efficient communication protocols [16,27]. While frequent data collection improves the accuracy of air quality measurements, it significantly increases the load on both the sensor network and the data processing center.

Efficient transmission scheduling is crucial for balancing monitoring reliability with communication costs [28,29]. In areas with dense sensor deployment, transmission frequency can be reduced for selected real-world nodes to minimize data redundancy. Conversely, in regions with rapidly fluctuating pollutant concentrations, schedules can prioritize higher transmission rates for more accurate tracking. For battery-powered sensors, longevity is a primary constraint, making energy conservation a key objective. One strategy to this end [28] involves neighboring nodes exchanging data exclusively upon detecting critical events. Dual-prediction strategies offer further efficiency: both the sensor and base station maintain a shared model to predict readings, triggering a transmission only when the actual measurement deviates beyond a predefined threshold. This selective communication paradigm significantly reduces redundant data transfer, thereby conserving energy and extending the operational network lifespan.

A principal strategy for reducing the substantial data volume transmitted in WSNs-based monitoring systems is the application of data compression. In [30], the authors describe a method for the mass deployment of IoT-based PM measurement devices. They demonstrate that compressed spatio-temporal data can reduce not only data transfer volumes but also energy consumption.

Previous studies have proposed various models for air quality analysis. For example, Markov process theory was used to model environmental pollution dynamics [31], and unsupervised learning was used for particle clustering [32]. In [33], the DL technique is used to predict air quality, using the EM algorithm to impute missing data. The authors conclude that approaches based on training datasets are insufficient.

The performance optimization of environmental monitoring systems is a widely researched field, with methodologies spanning various approaches [29,34]. The authors of [34] focus on optimizing sensor network design for pollution monitoring, specifically to identify atmospheric carbon dioxide hotspots. Their analysis of LoRaWAN-based wireless sensor networks employs a combined modeling and physical implementation approach, evaluated using packet loss metrics. Shifting to network design under uncertainty, the paper [29] tackles the challenge of designing AQM networks in coal ports, considering operational efficiency and uncertain wind conditions. Their proposed method formulates the deterministic AQM network design as a maximum-weight location problem, solving it with a progressive coverage model that incorporates a cooperative strategy.

The paper [35] presents a fog-enabled AQM system that integrates IoT, fog computing, and deep learning to improve monitoring accuracy and forecasting efficiency. While accurate particulate matter measurement traditionally requires expensive equipment, recent research demonstrates promising low-cost alternatives [36,37,38]. The literature thus clearly demonstrates the necessity for affordable yet effective monitoring, a challenge where machine learning methods are increasingly essential.

Thus, this paper advances the state-of-the-art by introducing the first EM clustering framework for air quality sensor networks. The proposed probabilistic approach uses soft cluster membership to simultaneously conserve network resources in clean zones and enhance monitoring in polluted areas. This way is inherently flexible, enabling adaptation to changing tasks and priorities.

## 3. Methodology

### 3.1. Expectation–Maximization Algorithm

The power of EM clustering lies in its probabilistic interpretation. Each data point does not belong rigidly to a single cluster but is instead described by a distribution of memberships. This reflects uncertainty in the data and aligns well with real-world situations where boundaries between groups are diffused rather than sharp. Moreover, EM provides a mathematically principled way to handle overlapping clusters, noisy measurements, and dynamic changes in the data distribution. These characteristics make it particularly well-suited for modeling air quality monitoring data, where pollutant concentrations exhibit smooth gradients, temporal fluctuations, and heterogeneous spatial distributions.

The EM algorithm is a maximum likelihood estimation framework for models involving latent (unobserved) variables. In clustering problems, the latent variable is the cluster membership of each data point, which is not directly observed. Unlike hard clustering methods that assign each data point to exactly one cluster, EM adopts a probabilistic model in which every observation may belong to each cluster with some probability. This approach is especially powerful when data are generated from a mixture of probability distributions, and the goal is to estimate both the mixture parameters and the soft cluster assignments.

Let us introduce the formalism of the EM algorithm, following the classical formulations presented in [39,40]. Let the dataset be $X=\{x_{1},x_{2},\cdots ,x_{N}\}$, and assume the data are drawn from a mixture of K distributions, where the probability mass function (pmf) or probability density function (pdf) of the k-th distribution is denoted by $f(x|\theta_{k})$. Thus, each distribution is parameterized by $\theta_{k}$ (individual parameter or set of parameters), and each cluster *k* has a mixing proportion $\pi_{k}$, with

(1) $$\sum_{k=1}^{K}\pi_{k}=1$$

The pdf/pmf of the mixture model is

(2) $$p\left(x_{i}|\Theta \right)=\sum_{k=1}^{K}\pi_{k}f(x_{i}|\theta_{k})$$

where $\Theta =\left\{\pi_{1},\pi_{2},\ldots ,\pi_{K},\theta_{1},\theta_{2},\ldots ,\theta_{K}\right\}.$

The notion of cluster membership is formalized through the introduction of a set of latent variables $Z=\{z_{1},z_{2},\cdots ,z_{N}\}$, one for each observation. Each $z_{i}$ is a *K*-dimensional binary random vector indicating which component generated the corresponding data point $x_{i}$. This vector uses a one-hot encoding, meaning

(3) $$z_{i,k}=\left\{\begin{array}{cc} 1, & if\;x_{i}\;belong\;to\;class\;k\\ 0, & otherwise \end{array}\right.$$

Assuming the data points are independent and identically distributed, the complete-data likelihood for the full dataset is then

(4) $$L\left(X,Z|\Theta \right)=\prod_{i=1}^{N}\prod_{k=1}^{K}{\left(\pi_{k}f(x_{i}|\theta_{k})\right)}^{z_{i,k}}$$

For computational simplicity, the model parameters are estimated by maximizing the expected value of the complete-data log-likelihood function:

(5) $$L\left(\Theta \right)=\ln L(X,Z|\Theta )=\sum_{i=1}^{N}\sum_{k=1}^{K}z_{ik}(\ln\pi_{k}+\ln f(x_{i}|\theta_{k}))$$

Since the latent variables $z_{ik}$ are unobserved, the function $L\left(\Theta \right)$ cannot be optimized directly. Instead, the EM algorithm maximizes its expected value, taken with respect to the posterior distribution of *Z* given the observed data *X* and the current parameter estimates $\hat{\Theta }$. Thus, the EM algorithm proceeds iteratively in two steps as follows.

- E-step (Expectation):
  Compute the posterior probabilities, often referred to as responsibilities, that each data point belongs to each cluster, given the current parameter estimates $\hat{\Theta }$. This responsibility, denoted $\gamma_{i,k}$, is the conditional expectation of the latent variable $z_{i,k}$ given the observed data and the current parameters:
  (6) $$\gamma_{i,k}=E\left(z_{i,k}|x_{i},\hat{\Theta }\right)=P\left(z_{i,k}=1|x_{i},\hat{\Theta }\right)$$
  An application of Bayes’ theorem provides the closed-form expression for the posterior responsibility, quantifying the probability that component *k* generated observation $x_{i}$:
  (7) $$\gamma_{i,k}(\hat{\Theta })=\frac{{\hat{\pi }}_{k}f(x_{i}|{\hat{\theta }}_{k})}{\sum_{j=1}^{K}{\hat{\pi }}_{j}f(x_{i}|{\hat{\theta }}_{j})}$$
  These probabilities express the degree of membership of point $x_{i}$ in cluster k. Each point is thus softly assigned to all clusters, with weights summing to 1 across clusters. This expression can be further simplified by substituting into it a probability mass (or density) function of practical interest. Next, since $L\left(\Theta \right)$ is a function of the unobserved latent variables Z, it is necessary to consider its expectation conditional on the observed data X and current parameter estimates, $\hat{\Theta },$ which defines the *Q*-function:
  (8) $$Q\left(\Theta ,\hat{\Theta }\right)=E_{Z|X,\Theta }\left[L\left(\Theta \right)\right]=\sum_{i=1}^{N}\sum_{k=1}^{K}\gamma_{i,k}(\hat{\Theta })(\ln\pi_{k}+\ln f(x_{i}|\theta_{k}))$$
- M-step (Maximization):
  The M-step involves maximizing the *Q*-function, computed in the previous E-step, with respect to the model parameters Θ to obtain an updated estimate:
  (9) $$\Theta^{new}=\arg\underset{\Theta }{\max}Q\left(\Theta ,\hat{\Theta }\right)$$
  The *Q*-function can be separated into two independent parts: one relating to mixture weights and the other to the parameters of probability distributions. This separation allows us to address each optimization problem individually. Therefore, taking (1) into account and applying the method of Lagrange multipliers, we derive the update rules for the mixture parameters:
  (10) $$\pi_{k}^{new}=\frac{1}{N}\sum_{i=1}^{N}\gamma_{i,k}\;\;\;\;\;\forall k\in \left\{1,2\ldots K\right\}.$$
  The update rules for the distribution parameters are derived by maximizing the corresponding term of the *Q*-function:
  (11) $$\theta_{k}^{new}=\arg\underset{\theta_{k}}{\max}\sum_{i=1}^{N}\gamma_{i,k}\ln f(x_{i}|\theta_{k}),\;\;\;\forall k\in \left\{1,2\ldots K\right\}.$$

In other words, the parameters of each component distribution are re-estimated by weighted maximum likelihood, where the weights are the posterior probabilities $\gamma_{i,k}$. The E-step and M-step are alternated until convergence, typically measured by changes in the log-likelihood function or in the parameter set Θ. Furthermore, an iteration limit can be imposed with the goal of keeping the algorithm’s runtime within acceptable bounds. The algorithm is guaranteed to converge to at least a local maximum of the likelihood function.

### 3.2. Model Specification

In line with our previous work [16], we consider air pollution monitoring through a sensor network capable of mobility. A mobile air quality sensor traverses a geographic region containing both areas of normal background air quality and zones with elevated pollution levels. The sensor is equipped to detect a specific pollutant and generates a message upon each significant detection event. The message generation process is modeled as a Poisson process, where the transmission rate is a function of the sensor’s location. Specifically, the message generation rate $\lambda_{1}$ is low when the sensor is outside the polluted zone, but it switches to a higher rate $\lambda_{2}$ ($\lambda_{1}<\lambda_{2}$) upon entering the polluted area. The Poisson distribution is a foundational model for data transmission and event count analysis in diverse systems, including communication networks. For example, in a typical scenario, the time to detect a critical event with a reusable mobile air quality sensor follows an exponential distribution [16]. The parameter μ of this distribution is determined by the specific characteristics of the sensor, including its mobility and performance. Consequently, the number of critical events detected within a fixed time interval, *T*, is described by a Poisson distribution with a rate parameter λ=μT. The probability of observing exactly *t* events in this interval is given by the Poisson pmf:

(12) $$P\left(\xi =t\right)=\frac{\lambda^{t}}{t!}e^{-\lambda }$$

here ξ is a random variable representing the number of events.

Analyzing the frequency of specific events, such as air pollution levels above a safety threshold, is a common approach in research. This count-based process is effectively modeled with a Poisson distribution, which is well-suited for rare events like hazardous pollution episodes. The logic behind this mirrors a standard method in environmental epidemiology. Emergency room visits for respiratory reasons are also counting processes, and their nature is very similar to the nature of hazardous pollution events [41,42]. The suitability of the Poisson model for air pollution exceedances is directly confirmed by works using real air quality data [43,44].

To complete the picture, we turned to real-world data. Data were collected by a sensor installed at road level in a highly polluted urban area in Italy [45], measuring the concentration of benzene (C_6_H_6_(GT)), an established air pollutant and human carcinogen. After removing missing values, the dataset contained 8991 records, with measured concentrations ranging from 0.1 to 63.7 µg/m^3^. We performed all calculations in this paper using Python (Version 3.11.13) and NumPy (Version 2.3.3). The descriptive statistics of the dataset are summarized in Table 1.

A threshold value was defined as 35% below the maximum recorded concentration, and the number of threshold exceedances was calculated for each consecutive 9 h interval. The Kolmogorov–Smirnov (K-S) test was applied to evaluate whether the exceedance counts followed a Poisson distribution. The resulting K–S statistic (the supremum distance between the empirical and theoretical cumulative distributions) was 0.0112, with a corresponding *p*-value of 0.998, indicating an excellent fit to the Poisson distribution.

This pattern is consistent across other pollutants. This trend is exemplified by non-methane hydrocarbons (NMHC), a key precursor to photochemical smog and thus a critical pollutant for this analysis. The accompanying descriptive statistics for this pollutant are provided in Table 2 for context. The K-S test, yielding a statistic of 0.0068 and a *p*-value of 1.0, demonstrates an excellent fit to the Poisson distribution. This statistical robustness of the NMHC concentration pattern holds regardless of specific geographical or meteorological conditions, as its primary source is localized vehicular traffic, whose emission profiles remain relatively constant.

The use of the Poisson pmf allows the derivation of closed-form expressions for both the Expectation and Maximization steps of the EM algorithm. This analytical tractability eliminates the need for numerical optimization or other computationally intensive procedures. As a result, the algorithm achieves high computational efficiency and rapid convergence.

The proposed EM algorithm is specifically provided to isolate the underlying emission processes that are fundamental drivers of air pollution. This core focus allows us to bypass secondary influences. Statistical validation, both within this work and as extensively documented in the literature, confirms that the concentration data for a range of primary pollutants adhere to a Poisson distribution. This finding is critical, as it establishes the sole prerequisite for our model’s application: the EM algorithm is universally applicable to any dataset where the target variable conforms to a Poisson process, irrespective of sensor type or local environmental conditions. The model’s strength lies in its ability to decode the latent emission signature directly from concentration readings without requiring ancillary data. Therefore, this work deliberately establishes a foundational model that captures the core statistical nature of emissions. Subsequent enhancements, which may integrate geographic and meteorological variables, will build upon this robust, generalizable core to address more complex, site-specific dispersion forecasting.

### 3.3. Refinement of EM Clustering

In the context of air quality monitoring, we consider the problem of distinguishing between regular background activity and air pollution events based on the number of alerts, $x_{i}$, recorded by a sensor over a fixed time interval. The observed dataset X, represents the count of alerts from all sensors in the monitoring area. We model these data as a mixture of two Poisson distributions, where the first component (k=1), parameterized by $\lambda_{1}$, models the low-rate Poisson process of normal background activity, and the second component (k=2), parameterized by $\lambda_{2}$ (where $\lambda_{2}\;$>$\;\lambda_{1}$), models the high-rate process of alert generation characteristic of a pollution event. This approach accounts for the fundamental uncertainty in attributing any individual observation with a high count to either a rare extreme value in the normal state or to a genuine pollution incident. The primary goal of applying the EM algorithm is to compute the responsibility for each sensor reading based on the estimated parameters (the mixing probabilities π,1−π and the rates $\lambda_{1}$, $\lambda_{2}$) thereby allowing each sensor’s reading to be probabilistically classified as either originating from a “polluted zone” or from the “normal state.” The properties of the Poisson distribution allow the update equations for the EM algorithm to be derived in a closed analytical form.

Therefore, we consider a mixture of two Poisson distributions parameterized by a mixing probability π, such that an observation belongs to class 1 with probability π and to class 2 with probability 1−π. Following the structure of the general mixture model in (2), the specific probability mass function for a single data point $x_{i}$ under a two-component Poisson mixture model is defined as

(13) $$p\left(x_{i}|\pi ,\lambda_{1},\lambda_{2}\right)=\pi \frac{{\left(\lambda_{1}\right)}^{x_{i}}}{x_{i}!}e^{-\lambda_{1}}+\left(1-\pi \right)\frac{{\left(\lambda_{2}\right)}^{x_{i}}}{x_{i}!}e^{-\lambda_{2}}$$

where $\Theta =\left\{\pi ,1-\pi ,\lambda_{1},\lambda_{2}\right\}$.

Substituting the Poisson probability mass function (12) into the responsibility formula and simplifying, we obtain the responsibility of cluster 1

(14) $$\gamma_{i,1}=\frac{\hat{\pi }e^{-{\hat{\lambda }}_{1}}{\left({\hat{\lambda }}_{1}\right)}^{x_{i}}}{\hat{\pi }e^{-{\hat{\lambda }}_{1}}{\left({\hat{\lambda }}_{1}\right)}^{x_{i}}+(1-\hat{\pi })e^{-{\hat{\lambda }}_{2}}{\left({\hat{\lambda }}_{2}\right)}^{x_{i}}}$$

The responsibility of cluster 2 is consequently

(15) $$\gamma_{i,2}=1-\gamma_{i,1}$$

From a computational perspective, it is methodologically advantageous to structure the calculations in the following manner:

(16) Ii=1γi,1=1+1π^−1expxi lnλ^2λ^1+λ^1−λ^2

The inverse responsibility calculation is numerically more stable because it avoids underflow errors that arise when directly processing extremely small probability values. It structures the computation to work with larger, more manageable numbers instead of perilously tiny ones. This method is also more computationally efficient as it reduces the number of complex exponential calculations required per data point. The resulting speedup can be crucial for processing large datasets effectively in real time.

The performance of the EM algorithm is highly sensitive to its initial parameter values [46]. In our case, if the initial values for the rate parameters $\lambda_{1}$ and $\lambda_{2}$ are identical, the model enters a symmetric state from which it cannot escape. This leads the algorithm to converge immediately to a degenerate solution where the parameter estimates for both components remain equal. Consequently, the model fails to recover the underlying mixture structure. To ensure a robust start, the initial values of Poisson distribution parameters are instead derived from the empirical data, *X*, with the smaller parameter set to the dataset’s lower quartile and the larger one to the upper quartile.

Within the framework of the EM algorithm for a two-class mixture model, the computation defined by Formula (10) reduces to

(17) $$\pi^{new}=\frac{1}{N}\sum_{i=1}^{N}\gamma_{i,1}.$$

The optimization problem (11) for finding parameter estimates of the distribution in this case reduces to the form:

(18) $$\lambda_{k}^{new}=\arg\underset{\lambda_{k}}{\max}\sum_{i=1}^{N}\gamma_{i,k}(x_{i}\ln\lambda_{k}{-\lambda }_{k}-\ln\left(x_{i}!\right)),\;k=1,2.$$

The term $\ln\left(x_{i}!\right)$ can be safely ignored in the optimization problem because it is a constant additive term with respect to the model parameters $\lambda_{k}$, and, therefore, its removal does not change the location of the extremum of the objective function. Therefore, to find $\lambda_{k}^{new}$ we maximize the following function:

(19) $$\tilde{Q}(\lambda_{k})=\sum_{i=1}^{N}\gamma_{i,k}(x_{i}\ln\lambda_{k}{-\lambda }_{k})$$

Set the derivative equal to zero to find the critical point:

(20) $$\frac{\partial \tilde{Q}}{\partial \lambda_{k}}=\sum_{i=1}^{N}\gamma_{i,k}\left(\frac{x_{i}}{\lambda_{k}}-1\right)=0$$

Solving this equation yields the update rule for the parameter:

(21) $$\lambda_{k}^{new}=\frac{\sum_{i=1}^{N}\gamma_{i,k}x_{i}}{\sum_{i=1}^{N}\gamma_{i,k}},\;\;k=1,2.$$

Let us check the second derivative to confirm that this critical point is a maximum.

(22) $$\frac{\partial^{2}\tilde{Q}}{\partial \lambda_{k}^{2}}={-\left(\frac{1}{\lambda_{k}}\right)}^{2}\sum_{i=1}^{N}\gamma_{i,k}x_{i}<0\;\;\;\forall \lambda_{k}$$

A negative second derivative at the critical point confirms a maximum. The second derivative is always negative (unless all $\gamma_{i,k}$ or $x_{i}$ are zero, which is a degenerate case). This conclusively proves that the obtained critical point is a global maximum for the function $\tilde{Q}(\lambda_{k})$ with respect to $\lambda_{k}$.

For completeness, we provide a compact pseudocode of the EM clustering algorithm used (Algorithm 1).

While the EM clustering algorithm does not have a definitive, universally optimal stopping rule, convergence is typically assessed by monitoring the relative increment in the observed data’s log-likelihood between iterations. The algorithm terminates once this change falls below a specified threshold, which indicates parameter stabilization near a local maximum. Additionally, a hard limit on the number of iterations can be set to prevent unnecessary computations should convergence be slow.
**Algorithm 1. **EM Clustering.1:  **Input:** Dataset ***X***, stop_rule 2:  **Initialize: ** 3:   $\;\lambda_{1}\;$← first quartile of ***X*** 4:   $\;\lambda_{2}\;$← third quartile of ***X*** 5:          π  ← 0.5                                  # *mixing proportion for cluster 1* 6:  **Repeat** until stop_rule:        7:    For each *i*:*                            # E-step* 8:      Calculate $I_{i}$** ** 9:      $\gamma_{i,1}=1/I_{i}$ 10:    $\gamma_{i,2}=1-\gamma_{i,1}$     11:       Update π                                 *# M-step* 12:       Update $\lambda_{1},\lambda_{2}$ 13:       Prepare for convergence check 14:  **Check** stop_rule 20:  **Return** $\pi ,\lambda_{1},\lambda_{2},\gamma_{i1},\gamma_{i2}$          # Output


## 4. Performance Analysis

This section presents the results of a simulation-based performance evaluation of the EM algorithm for the soft clustering of air sensors. The objective is to evaluate the algorithm’s ability to correctly classify sensors into “normal air” or “pollution” clusters, under the assumption that the rate of detection for events of interest differs for each case. Our experimental procedure involves generating separate samples for two air quality scenarios using pseudorandom number generators for the Poisson distribution with different parameters. To generate a sample corresponding to observations in the normal situation, we use parameter $\lambda_{1}$, and for a sample corresponding to air pollution, we use parameter $\lambda_{2}$, where $\lambda_{1}<\lambda_{2}$. These samples are combined and randomly shuffled to create a dataset with an unknown underlying structure, simulating data from a real sensor network. The EM algorithm is applied to this combined sample to estimate the mixture parameters and calculate the cluster membership probabilities (responsibilities). To evaluate the algorithm’s performance, the results are analyzed separately for each of the original samples.

During simulation runs, a seed was initialized using pseudorandom integers uniformly distributed between 0 and 1000. Training was terminated when the convergence threshold of 10^−7^ was reached.

**Figure 1:**
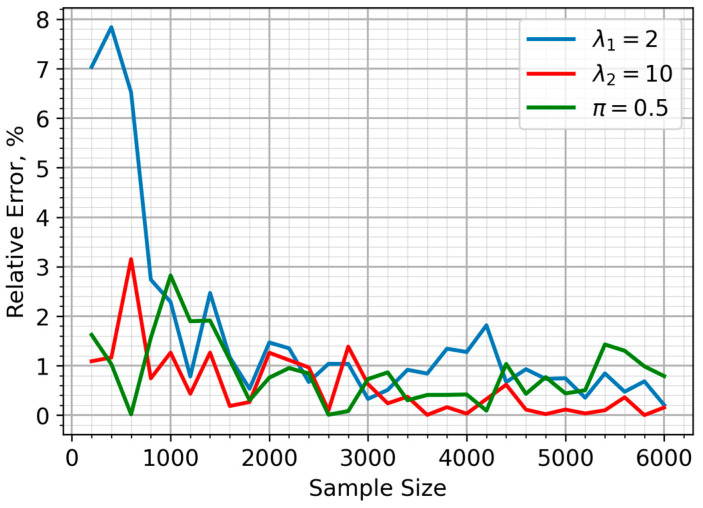
Relative error of parameter estimates for the mixture model.

The EM clustering algorithm demonstrates a strong ability to accurately estimate the underlying mixture components: $\lambda_{1},\lambda_{2}$ and π. As illustrated in Figure 1, the relative error for each parameter remains low, generally not exceeding a few percent across the tested sample sizes. However, as the sample size grows, the error does not follow a monotonic decreasing trend but demonstrates fluctuations.

This non-monotonic behavior is expected because the EM algorithm converges to a local maximum of the likelihood function. The specific random sample drawn for a given size can slightly bias the initial conditions or the convergence path, leading to minor variations in the final estimates. Consequently, while larger samples provide more stable estimates on average, the stochastic nature of both the data generation and the EM optimization process results in natural fluctuations in accuracy.

**Figure 2:**
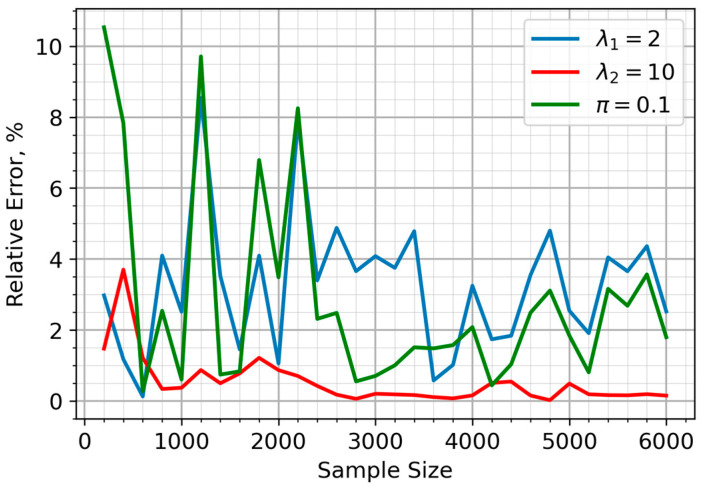
Relative errors of parameter estimate for the mixture model when 90% of sensors are located in the air pollution zone.

**Figure 3:**
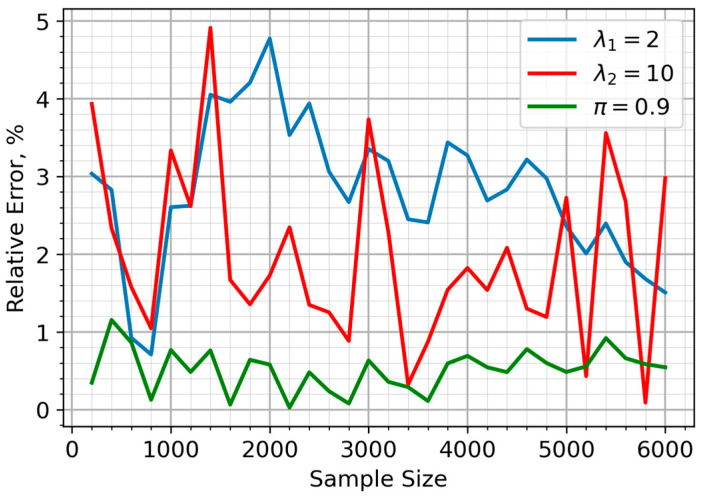
Relative errors of parameter estimate for the mixture model when 10% of sensors are located in the air pollution zone.

In Figure 1, it is assumed that the sensors have an equal probability of being in either the clean or polluted air zones (π=0.5). The relative error of the parameter estimates, for the situation in which 90% of sensors are within the air pollution zone (π=0.1), is illustrated in Figure 2. The proposed approach yielded a highly accurate and stable estimate for the λ_2_ parameter, corresponding to sensor data from the pollution zone. While the accuracy of the λ_1_ estimate decreased compared to the equal sample size scenario, this only impacted a minor portion of the sample (10%). Figure 3 depicts the case where the majority of sensors (90%) are in unpolluted zones (π=0.9). As expected, the parameter estimates show noticeable fluctuations in accuracy, though these consistently stay within a 5% margin.

In all cases considered, the EM algorithm correctly assigns sensors to their corresponding class with near certainty (responsibilities are very close to 1).

**Figure 4:**
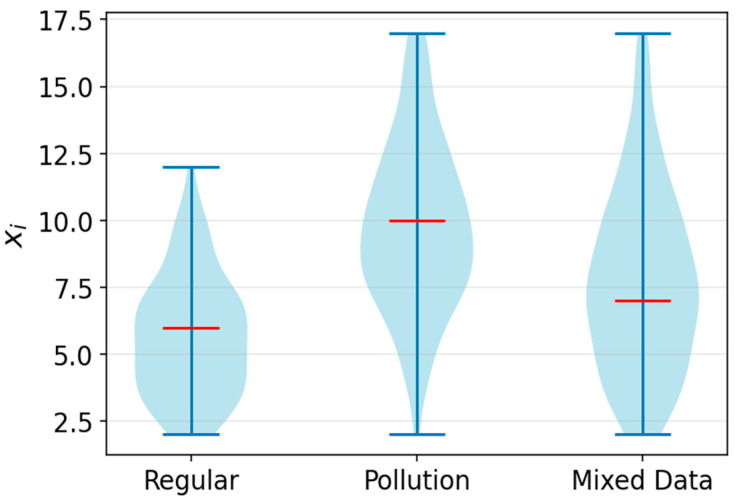
Visualization of the dataset for a challenging scenario.

**Figure 5:**
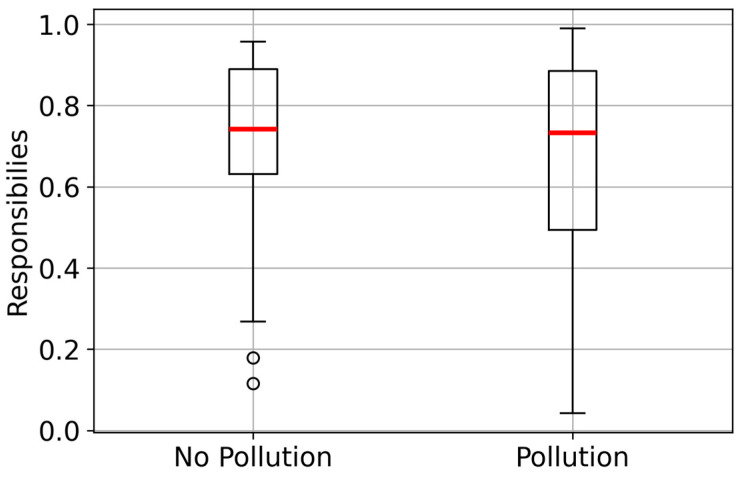
Cluster membership probabilities from the EM algorithm.

Let us consider an extreme scenario with a modest sample size (*N* = 200) and relatively close detection intensities ($\lambda_{1}=6,\lambda_{2}=10$). The dataset generated for this situation is presented in Figure 4 as a violin plot. This violin plot illustrates the smoothed density of data distribution, where its width shows the frequency of values, and the inner red line indicates the median. The result of EM clustering for π=0.5 is shown in Figure 5. A shift in π improves the estimation accuracy for the predominant subsample.

In this scenario, unlike hard clustering approaches (e.g., k-means), which would disable over 30% of sensors in the pollution zone, probabilistic clustering enables the involvement of all these sensors in intensive and detailed air pollution monitoring via parameterized data transmission policies. Even a simple activation policy, triggering intensive operation or a switch to energy-saving mode when a sensor’s probability exceeds a 0.5 threshold, ensures that a significant majority of sensors are correctly assigned an operational state commensurate with their true status. Dynamic resource management based on responsibility information ensures almost full activation of sensors in hazardous regions at the expense of temporary acceptance of additional short-term costs. Conversely, the same framework allows for a substantial reduction in monitoring intensity and data transmission volume within safe zones. This adaptive strategy ensures that limited resources are allocated to activities of the highest operational relevance. Although this may result in lower-resolution monitoring in non-critical areas, it substantially enhances overall cost-effectiveness. Moreover, it addresses an inherent flaw of hard clustering, where the misclassification of a subset of sensors can create critical blind spots that lead to the loss of essential data from contaminated regions and consequently to high economic and societal costs.

**Figure 6:**
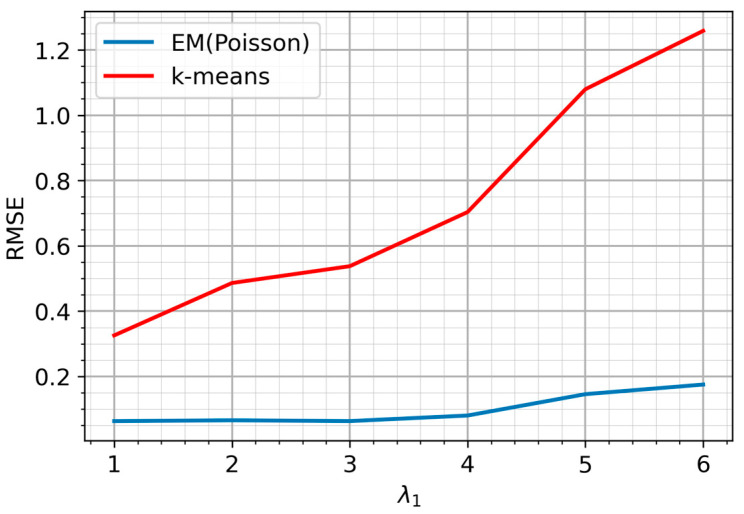
RMSE of Parameter Estimates for EM Clustering and k-means.

Next, we compare the estimates derived from the proposed EM algorithm with those obtained through k-means clustering using Root Mean Square Error (RMSE). Although the parameters of the Poisson mixture ($\lambda_{1}$ and $\lambda_{2}$) and k-means centroids are fundamentally different in nature, representing probabilistic parameters and geometric centers, respectively, this comparison is methodologically justified in the context of this paper. Since the data are generated from a known Poisson mixture, the k-means centroids can be interpreted as empirical estimates of the distribution means. Thus, evaluating both methods via RMSE provides meaningful insight into their relative effectiveness at recovering the true data-generating parameters. Figure 6 presents the results of performance comparison with a fixed $\lambda_{2}=10$ and a varying $\lambda_{1}$.

The results demonstrate a superior performance of the EM algorithm over k-means. As expected, estimation accuracy for both methods degrades as $\lambda_{1}$ increases and the distributional dissimilarity diminishes. However, the EM algorithm’s performance degrades far more gradually. Its RMSE increases at a significantly slower rate than that of the k-means estimator.

To further demonstrate the advantages of the EM algorithm, we introduce a scenario with a penalty function. To convert the soft assignments of the EM algorithm into hard clusters, an observation i is assigned to class k if its responsibility $\gamma_{i,k}$ exceeds a chosen threshold h. This threshold is selectable to suit different application needs. The penalty function is defined as follows:

(23) $$f_{p}=N_{err}^{\left(1\right)}+{c\cdot N}_{err}^{\left(2\right)}$$

where $N_{err}^{\left(1\right)}$ is the number of misclassified sensors in the clean area, and ${c\cdot N}_{err}^{\left(2\right)}$ is the number of misclassified sensors in the polluted area. The cost of a false alarm in the clean zone (e.g., unnecessary transmission of redundant data) is normalized to 1. The coefficient c therefore represents the relative cost of a missed detection in the polluted area, which corresponds to the loss of valuable data.

**Figure 7:**
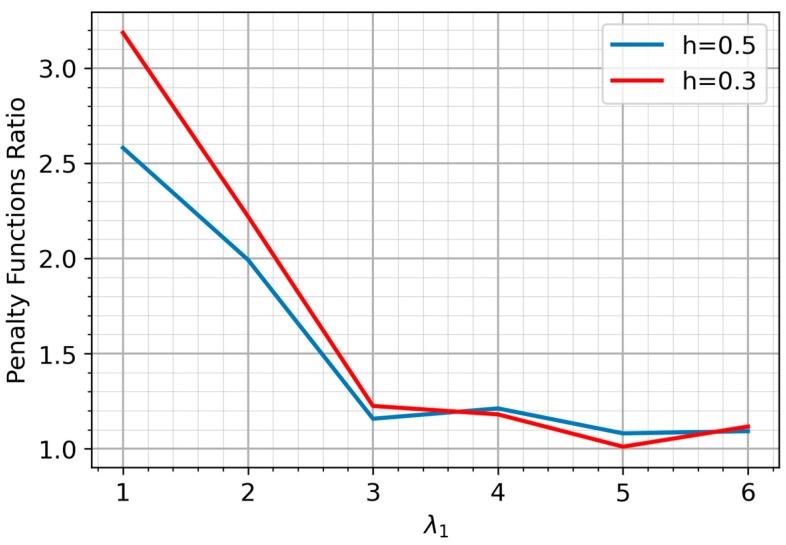
Ratio of k-means to EM Penalty Function.

Under an equal mixing coefficient (π=0.9), which represents an ideal scenario for k-means, the performance was evaluated by calculating the ratio of the k-means penalty value to that of the EM algorithm. The optimal threshold h is inversely related to the penalty weight c. We select a boundary value of c=1.2 to analyze the transition where both error types are equally penalized, and h∈{0.5;0.3}. Figure 7 presents the results of this comparison.

As expected, under typical high-pollution conditions, the EM algorithm significantly outperforms k-means across both threshold values, even with balanced cluster sizes. A more important finding is the inherent ambiguity in selecting an optimal threshold. This ambiguity reveals a key advantage of the EM-based approach: it provides flexibility to tailor the model to specific operational priorities.

The performance analysis reveals that the proposed EM clustering method is subject to certain limitations despite its overall effectiveness. Its performance can degrade when the underlying Poisson distributions exhibit significant overlap in their parameters, making the components difficult to distinguish. Furthermore, the method is sensitive to small sample sizes, which can lead to unstable parameter estimates. To reduce the inherent volatility of the EM algorithm when applied to small sample sizes, a reasonable strategy is to initialize the procedure from a set of random starting points, thereby protecting against spurious convergence to an unrepresentative local optimum and providing more reliable parameter estimates.

## 5. Conclusions

This paper addresses the critical challenge of data aggregation in large-scale air pollution monitoring networks, where the volume and stochastic nature of sensor data can lead to significant communication overheads. This paper investigates the application of unsupervised machine learning, a branch of artificial intelligence, for enhancing the performance of air quality monitoring systems. We proposed and validated a modified Expectation–Maximization algorithm for the soft clustering of sensors. By modeling sensor alert signals as a mixture of Poisson distributions, our method probabilistically distinguishes between normal background activity and pollution events, assigning each sensor a cluster membership probability. The power of this approach lies in its probabilistic foundation, which naturally handles the uncertainty and diffuse boundaries inherent in environmental data. Unlike hard clustering techniques, our model provides a nuanced view of the monitoring landscape, enabling the implementation of dynamic data transmission policies.

Simulation results confirm the high efficiency of this method, demonstrating that the cluster membership probability serves as a robust mechanism for controlling the fundamental trade-off between data redundancy and monitoring accuracy. As expected, EM clustering produces an almost perfect assignment of responsibilities when the underlying Poisson distributions are well separated. Surprisingly, even in the more challenging scenario of closely spaced intensity parameters, the algorithm correctly identifies the true cluster with a probability exceeding 0.5 in more than 75% of cases. Furthermore, in over half of these cases, the correct cluster is identified with a probability greater than 0.75. The results provided can be used to open promising avenues for future research into more sophisticated, self-organizing data aggregation protocols for environmental sensing and other distributed monitoring applications.

## Figures and Tables

**Table 1:** Descriptive statistics for benzene concentration.

mean	10.083105
std	7.449820
min	0.100000
25%	4.400000
50%	8.200000
75%	14.000000
max	63.700000

**Table 2:** Descriptive statistics for non-methane hydrocarbons concentration.

mean	218.811816
std	204.459921
min	7.000000
25%	67.000000
50%	150.000000
75%	297.000000
max	1189.000000

## Data Availability

The original contributions presented in this study are included in the article. Further inquiries can be directed to the corresponding author.

## Abbreviations

The following abbreviations are used in this manuscript.

AQM	Air Quality Monitoring
WSNs	Wireless sensor networks
IoT	Internet of things
EM	Expectation–maximization
pmf	Probability mass function
pdf	Probability density function
ML	Machine learning
DL	Deep learning
RMSE	Root mean square error
